# Novel Synthesis of IMC-48 and Affinity Evaluation with Different i-Motif DNA Sequences

**DOI:** 10.3390/molecules28020682

**Published:** 2023-01-10

**Authors:** Florian Berthiol, Joseph Boissieras, Hugues Bonnet, Marie Pierrot, Christian Philouze, Jean-François Poisson, Anton Granzhan, Jérôme Dejeu, Eric Defrancq

**Affiliations:** 1 Department of Molecular Chemistry (DCM), CNRS, UMR 5250, Université Grenoble-Alpes, 38000 Grenoble, France; 2 CNRS UMR9187, INSERM U1196, Institut Curie, PSL Research University, F-91405 Orsay, France; 3 CNRS UMR9187, INSERM U1196, Université Paris Saclay, F-91405 Orsay, France; 4 FEMTO-ST Institute, CNRS UMR-6174, Université de Bourgogne Franche-Comté, F-25000 Besançon, France

**Keywords:** i-motif DNA, bio-layer interferometry, circular dichroism, affinity, IMC-48, DNA ligands, steroid derivatives

## Abstract

During the last decade, the evidence for the biological relevance of i-motif DNA (i-DNA) has been accumulated. However, relatively few molecules were reported to interact with i-DNA, and a controversy concerning their binding mode, affinity, and selectivity persists in the literature. In this context, the cholestane derivative **IMC-48** has been reported to modulate *bcl-2* gene expression by stabilizing an i-motif structure in its promoter. In the present contribution, we report on a novel, more straightforward, synthesis of **IMC-48** requiring fewer steps compared to the previous approach. Furthermore, the interaction of **IMC-48** with four different i-motif DNA sequences was thoroughly investigated by bio-layer interferometry (BLI) and circular dichroism (CD) spectroscopy. Surprisingly, our results show that **IMC-48** is a very weak ligand of i-DNA as no quantifiable interaction or significant stabilization of i-motif structures could be observed, stimulating a quest for an alternative mechanism of its biological activity.

## 1. Introduction

**Figure 1:**
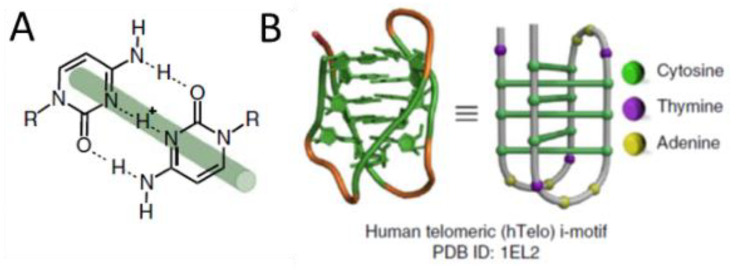
(**A**) Hemi-protonated CC base pair. (**B**) Schematic representation of the structure of the telomeric i-DNA.

The double-helical structure of DNA in which two antiparallel strands are held together through canonical AT and GC base pairing has been resolved over half a century ago. However, beyond double helical-based structures, the past decades have brought accumulating evidence of the existence of more complex nucleic acid structures, including four-stranded G-quadruplexes and i-motifs [1,2]. i-Motifs of DNA (hereafter, i-DNA), known in vitro for nearly three decades [3], are unusual four-stranded structures, in which cytosines are intercalated via a stack of hemi-protonated CC base pairs (CH^+^:C) (Figure 1A,B) [4]. Some of these structures have been well characterized in vitro and, because i-DNA may mirror other four-stranded G-rich structures (G-quadruplexes, or G4) present in gene promoters or at telomeres, their biological relevance is being investigated [2]. By using the Quadparser algorithm, more than 5000 putative i-motif forming sequences have been found in the human genome. Interestingly, more than 600 were found to be located within promoter sequences of genes such as *c-myc*, *bcl-2* or *c-kit* [5]. Other studies have proposed that i-DNA is involved in the regulation of transcription [6] and in the integrity of telomeric DNA [7]. A major characteristic of i-DNA is the strong pH-dependency of its stability and formation. Indeed, i-DNA structures are typically observed in vitro at acidic pH, a particularity that has cast doubt on their existence in cellulo. However, two independent studies have recently demonstrated the stability of exogenous i-DNA structures in human cells (through in cellulo NMR spectroscopy) [8] as well as the presence of endogenous i-DNA in the nuclei of human cells (through immunofluorescence using an antibody *i-Mab* raised against the i-motif) [9].

In this context, the development of i-motif binders (or ligands) is of great interest in view of their potential use as selective chemical probes. However, in contrast to a vast number of G4 ligands, relatively few molecules have been reported to interact with i-DNA, and a controversy concerning their binding mode, affinity, and selectivity still persists in the literature [10,11]. The main challenges in this regard are the strong pH-dependency, flexibility, and polymorphism of i-DNA, that introduce potential bias into screening methods. Indeed, low-pH conditions that are required for the formation of i-DNA can lead to the protonation of many ligands, strongly increasing their non-specific nucleic acid binding. This point is of high importance because the use of selective small molecules to study the biological functions of such structures is essential. As a matter of example, a recent report claimed the selective stabilization of i-DNA in cellular context by ellipticine (NSC71795), a well-known (since 1975) DNA intercalator and topoisomerase II poison [12]. To date, a number of promiscuous DNA-binding molecules, including TMPyP4 [13,14], mitoxantrone [15], [Ru(phen)_2_dppz]^2+^ [16], berberine [17], and others such as G4 ligands PhenDC3, BRACO-19 and PDS, have been reported as putative i-DNA ligands. To address this controversy, in a recent study, we have investigated the interaction of these previously reported i-DNA “ligands” with folded or unfolded i-DNA in acidic (pH 5.5) and near-neutral (pH 6.5) conditions, respectively. We have demonstrated that most of them do not display a selective interaction with the i-DNA structure nor are able to promote its formation [18]. Likewise, Gargallo and coll. also demonstrated that berberine binds equally well to the folded telomeric i-DNA and the unfolded cytosine-rich strand, without producing any stabilization of the i-DNA structure [19].

In 2014, by using an isothermal FRET high-throughput screening assay, Hurley and coll. identified the cholestane derivative **IMC-48** able to interact with the i-motif formed from *bcl-2* sequence [20]. The characterization of the interaction of this compound with the target *bcl-2* i-DNA was performed through NMR and CD melting analysis. The authors concluded that **IMC-48** binds the central loop of *bcl-2* i-motif, and a *K_D_* value of 0.49 μM was estimated from the isothermal FRET assay. However, it should be noted that the increase of the melting temperature, ∆*T*_m_ (in CD melting experiments), was only of 1 °C, which could suggest a weak binding. Nevertheless, **IMC-48** has been shown to produce a clear effect on *bcl-2* gene expression in cells, with a significant up-regulation of the *bcl-2* gene and associated protein expression [20,21].

In this context, we report herein on the investigation of the recognition properties of **IMC-48** for different i-motif-forming DNA sequences by using bio-layer interferometry (BLI) and circular dichroism (CD)-based denaturation experiments. Bio-layer interferometry analysis (BLI) is an optical technique that has been recently implemented to study biomolecular interactions between small molecules and different DNA secondary structures [22,23,24,25]. A major advantage of BLI is that it allows the determination of the kinetic and thermodynamic parameters such as surface plasmon resonance (SPR), but without the problems associated with the SPR micro-fluidic system and variations in bulk contribution [26]. Furthermore, the original synthesis of **IMC-48,** reported almost 60 years ago by Tsatsas and Vassiliadou, was tedious and required nine steps [27]. Thus, we also report in this paper a novel synthesis of **IMC-48** which proves more efficient and requires only four steps. The structure of **IMC-48** produced by this novel route was unambiguously confirmed by X-ray crystallography.

## 2. Results and Discussion

### 2.1. Novel Synthesis of IMC-48

**Scheme 1:**
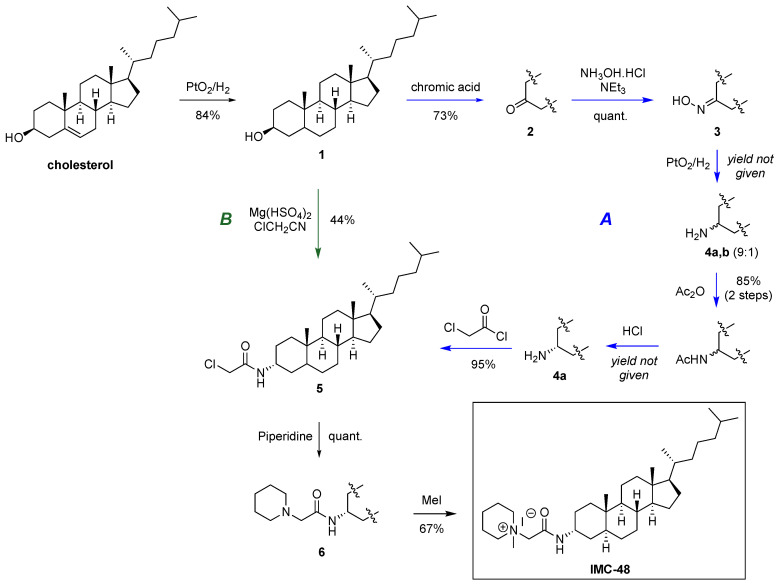
Synthesis of **IMC-48**: (**A**) synthesis from reference [25]; (**B**) new synthesis.

The previously reported synthesis of **IMC-48** (Scheme 1A, blue arrows) started by the reduction of cholesterol into cholestanol **1** using platinum oxide under hydrogen atmosphere [27]. Cholestanol **1** was then oxidized by chromic acid into cholestanone **2**, which was further transformed into cholestanone oxime **3**. The subsequent reduction of the oxime using platinum oxide under an hydrogen atmosphere afforded a 9/1 mixture of 3α- and 3β-aminocholestanes **4a,b**. This mixture was next acetylated in order to separate the diastereoisomers by alumina column chromatography. After deprotection, 3α-aminocholestane **4a** was reacted with chloroacetyl chloride to provide the key intermediate, chloroacetylaminocholestane **5**. The substitution of the chlorine atom by piperidine gave piperidinoacetylaminocholestane **6**, which was methylated with methyl iodide to afford **IMC-48**. In this initial report, the steps from aminocholestane **4** to **IMC-48** were mentioned, but without experimental details and no state-of-the-art compound characterization data. In particular, no experimental details regarding the reduction of cholestanone oxime **3** into aminocholestane **4** were provided. As attempts to reproduce this synthesis failed to give reliable results, we were motivated to develop a shorter and more convenient synthesis of the key intermediate, chloroacetylaminocholestane **5**.

**Figure 2:**
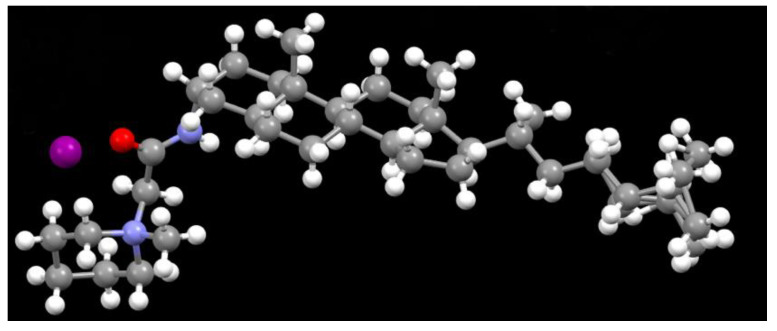
X-ray crystal structure of **IMC-48** synthesized by the new route.

In this context, we envisioned the possibility to directly transform the alcohol group of cholestanol **1** into the desired amide of **5,** based on the work of Salehi et al. who described the direct transformation of cyclohexanol into chloroacetylaminocyclohexane in only one step [28]. The method was thus adapted to transform in a single step cholestanol **1** into chloroacetylaminocholestane **5** (Scheme 1B, green arrow). Simply diluting cholestanol **1** in chloroacetonitrile in the presence of magnesium hydrogenosulfate directly afforded the amide **5** in 44% yield with an 85/15 diastereoisomeric ratio. Furthermore, the major stereoisomer could be isolated in pure form by recrystallization from ethyl acetate, affording crystals suitable for X-ray analysis. Crystal structure elucidation showed that the major diastereomer was the desired 3α isomer, in agreement with a reasonable axial attack of an intermediate carbocation (see the supporting information, Figure S6). Following the described remaining two steps, **IMC-48** was also isolated as single crystals, by slow evaporation of ethanol at room temperature. Its crystal structure, depicted in Figure 2, confirms unambiguously its structure and configuration. This straightforward alcohol to amide route thus allows the shortening of the initial reported synthesis by five steps (9 to 4), with a reliable procedure and easy purifications.

### 2.2. Evaluation by BLI of the Affinity of **IMC-48** for DNA Sequences

The affinity of **IMC-48** for *bcl-2* i-motif has been previously investigated using isothermal FRET assay by Hurley and coll. [20]. In this study, the effect of ligands was evaluated by monitoring the folding of the i-DNA, which is manifested through a FRET quenching of the double-labelled (5′-FAM/3′-BHQ1) *bcl-2* oligonucleotide in the presence of increasing concentrations of the ligand (0.5 to 5 molar equivalents). In the conditions of that study (50 mM Na cacodylate buffer, pH 5.8), the relative fluorescence variation at 520 nm (i.e., FAM emission) was less than 10%, and an equilibrium dissociation constant *K_D_* of around 490 nM was determined. However, it has been more recently shown that fluorophores typically used in the FRET assay may introduce strong biases into ligand interactions with i-DNA [29,30], namely by direct fluorophore–ligand interactions. Moreover, the authors have performed CD melting experiments and observed a very low effect of the ligand on thermal stability of *bcl-2* i-DNA (∆*T*_m_ = 1 °C), which appears rather surprising considering the reported sub-micromolar affinity.

We have thus decided to investigate the interaction of **IMC-48** with several DNA oligonucleotide sequences (Table 1). The evaluation of the binding of **IMC-48** was performed with the four sequences *HRAS*, *bcl-2*, *c-myc*, and the telomeric sequence *HTelo-C,* at two distinct pH conditions: at acidic pH of 5.5 at which the studied sequences are folded into i-DNA structures (as confirmed by the CD spectrum which displayed a positive peak at 286 nm and a negative peak at 262 nm, characteristic of the formation of the i-motif [31]), and at near-neutral pH of 7.4 at which they adopt a random-coil structure as evidenced by CD spectra featuring a low-intensity peak at 270–280 nm (see Figure S14 in the supporting information). Of note, the structure of **IMC-48** does not have protonable nitrogen atoms, and its charge (and hence non-specific DNA binding) should not depend on pH. Single stranded *ssDNA*, double-stranded DNA (hairpin *HP-GC*), and a mutant sequence of *DAP* [32] (*DAP-Mut*) were used as controls.

**Figure 3:**
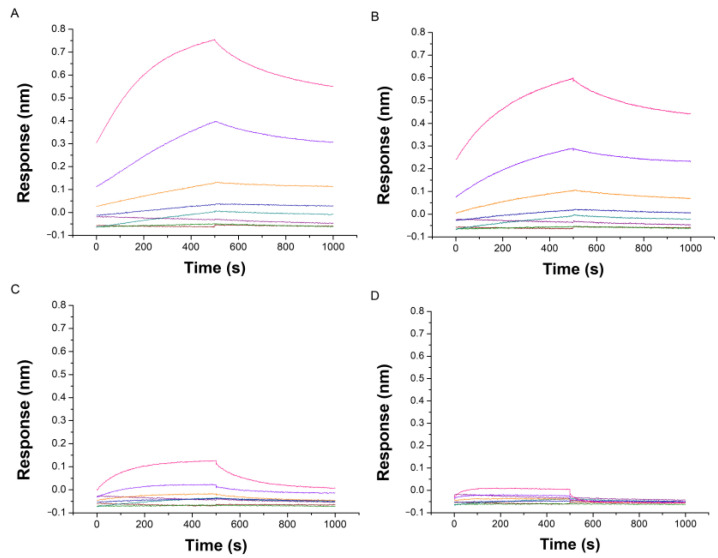
Sensorgrams recorded at pH 5.5 for the recognition of **IMC-48** with immobilized sequences: (**A**) *HRAS*; (**B**) *bcl-2*; (**C**) *Htelo-C*; and (**D**) *c-myc*. Concentration range: 0.1 (blue), 0.5 (brown), 1 (green), 5 (dark cyan), 10 (black), 25 (orange), 50 (violet), and 100 µM (pink). Buffer composition: 50 mM TrisHCl, 50 mM KCl, 2% DMSO and 0.05% *v/v* surfactant P20.

**Figure 4:**
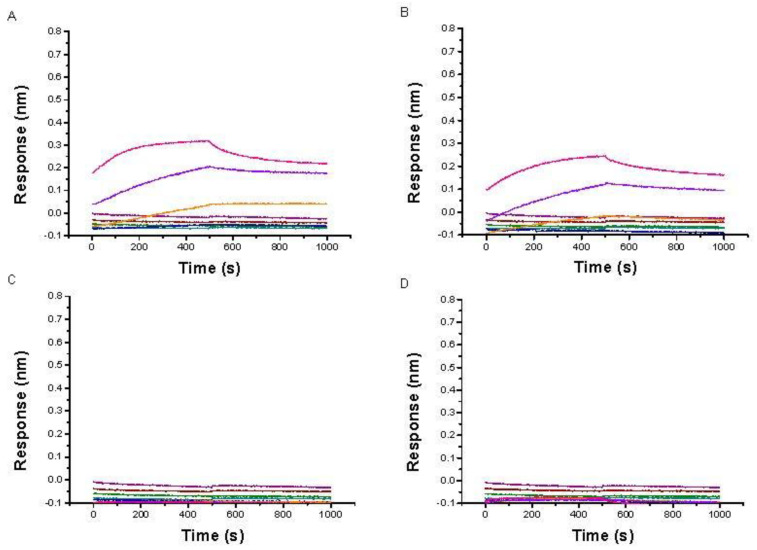
Sensorgrams recorded at pH 7.5 for the recognition of **IMC-48** with immobilized sequences: (**A**) *HRAS*; (**B**) *bcl-2*; (**C**) *HTelo-C*; and (**D**) *c-myc*. Concentration range: 0.1 (blue), 0.5 (brown), 1 (green), 5 (dark cyan), 10 (black), 25 (orange), 50 (violet), and 100 µM (pink). Buffer composition: 50 mM TrisHCl, 50 mM KCl, 2% DMSO and 0.05% *v/v* surfactant P20.

The interaction was first studied by using BLI. For this purpose, the different oligonucleotide sequences (Table 1) were biotinylated at their 3’-termini and anchored on the BLI sensors through biotin-streptavidin interactions. The sensors were next dipped into the solutions containing **IMC-48** at concentrations ranging from 100 nM to 100 µM. The sensors were not regenerated after each measurement and the obtained sensorgrams are depicted in Figure 3 and Figure 4 for pH 5.5 and pH 7.5, respectively. As shown in Figure 3B, **IMC-48** binds the *bcl-2* sequence in its folded form, as evidenced by the dose-dependent increase of the BLI response, which is in agreement with the previously reported results [20]. However, a visual observation clearly indicates that the affinity is far from the reported ~500 nM *K_D_* value. Indeed, the signals start to increase at a concentration between 10 and 25 µM (black and orange sensorgrams, respectively, in Figure 3B). Below these concentrations, the response is quasi-null. It should be noted that **IMC-48** also interacts with *HRAS* at similar concentrations (Figure 3A), whereas it does not bind *Htelo-C* and *c-myc* sequences in the tested concentration range (from 0.1 to 100 µM). The weak interaction observed with *bcl-2* and *HRAS* i-motif structures may be attributed to the formation of hairpins by these sequences, presumably acting as weak ligand binding sites, but we have not investigated it. It should be noted that the shape and the amplitude of the sensorgrams does not allow an exact determination of the *K_D_* values (examination of the Langmuir analyses in Figure S15 in the supporting information shows that the plateau value is not reached at the studied concentration). With regard to Figure 4A,B, the *K_D_* value could be estimated for the *HRAS* and *bcl-2* sequences, superior to 50 µM.

More surprisingly, when the experiments were performed at pH 7.5, i.e., in conditions in which the DNA sequences are not folded into an i-motif structure, we obtained results that were qualitatively similar to those obtained at pH 5.5 (Figure 4). Indeed, a weak affinity with *HRAS* and *bcl-2* sequences was observed while no recognition was detected with sequences *HTelo-C* and *c-myc*. The same behavior was already observed in our previous study using a panel of putative i-motif ligands which were found to not discriminate between folded and unfolded i-motif structures [18]. In summary, the results of BLI measurements indicate weak binding of **IMC-48** to *bcl-2* and *HRAS* sequences, both in acidic (favoring i-DNA) and neutral (disfavoring i-DNA) conditions, and the absence of interaction with *HTelo-C* and *c-myc* sequences, and they speak against a structure-selective binding to i-motifs.

It should be also noticed that **IMC-48** does not show a significant affinity for the control sequences single stranded *ssDNA*, double-stranded *dsDNA* (hairpin *HP-GC*), and the mutant sequence of *DAP* (see Figure S16 in the supporting information).

To confirm the obtained results of low binding of **IMC-48** to *bcl-2* sequence which are not consistent with the previous study, CD experiments were performed.

### 2.3. Circular Dichroism Studies

To further investigate the effect of **IMC-48** on i-DNA versus single-strand equilibria, we used three methods based on circular dichroism (CD) spectroscopy. First, CD spectra of the four DNA oligonucleotides were recorded in the absence and in the presence of **IMC-48** (20 µM), both in acidic (pH 5.5) and neutral (pH 7.2) conditions (Figure S17). In the case of *HRAS*, *bcl-2*, and *HTelo-C* (panels A–C), the shapes of CD spectra were not affected by the presence of **IMC-48**, neither in acidic (where all oligonucleotides demonstrated CD spectra typical of i-DNA) nor in neutral conditions (where all oligonucleotides demonstrated CD spectra typical of random-coiled structures), giving evidence of the absence of ligand-induced structural changes. In the case of *c-myc* (panel D), the addition of the ligand induced small changes in CD spectra, namely a decrease and a small red shift of the i-DNA-characteristic CD band at 285 nm at pH 5.5, and a small increase of the random-coil-characteristic band at 270 nm at pH 7.2. However, these changes alone were not sufficient to claim the binding of ligand to the *c-myc* i-DNA.

Next, to assess the potential stabilizing effect of **IMC-48**, thermal denaturation (or “CD-melting”) experiments were performed with the four DNA oligonucleotides in acidic conditions (pH 5.5) by recording the i-DNA-characteristic CD signal at 285 nm as a function of temperature. Notably, the presence of **IMC-48** (20 µM) led to only slight changes in the denaturation temperature of i-DNA (Table 2 and Figure S18 in the supporting information). In the case of *bcl-2,* the melting temperature was increased by 0.2 °C, a value which is inside the experimental error range (±1 °C in our settings), and even less than 1 °C as described in the original report, despite a two-fold higher concentration of the ligand [20]. For all other sequences, *T*_m_ values slightly decreased, again within or close to the experimental error range. In either case, the variations of melting temperature were too weak to suggest a significant impact on the stability of i-DNA despite a relatively high concentration of the ligand.

**Figure 5:**
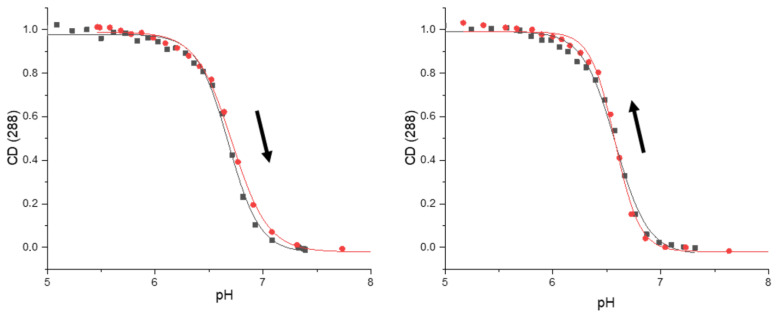
Representative pH-dependent denaturation/renaturation of i-DNA: normalized changes of ellipticity at 285 nm in the course of potentiometric titrations of *bcl-2* performed in the absence (black points and curves) and in the presence (red points and curves) of 20 µM **IMC-48**. For the detailed conditions, cf. Table 2 legend. (**Left**) alkali (denaturing) titration; (**right**) acid (renaturing) titration. The arrows indicate the denaturing/renaturing processes.

Next, we evaluated the effect of the ligand on chemical denaturation of i-DNA observed upon increasing pH of the medium, as it was suggested that ligands or crowding agents that bind to i-DNA could stabilize them against alkali-induced unfolding (similarly to what is typically observed in thermal denaturation experiments) [33,34]. To this end, pH_T_ values of the oligonucleotides, characterizing the stability of i-DNA, were determined by measuring the CD signal at 285 nm as a function of pH during alkali (denaturing) and acid (renaturing) titrations, performed in the absence and in the presence of **IMC-48** (Figure 5, Table 2). In agreement with the results of thermal denaturation experiments, the presence of the ligand had only a marginal effect on pH_T_ of the studied sequences, suggesting that the ligand is unable to stabilize i-DNA to an extent that would be sufficient to render it persistent at physiological conditions.

## 3. Conclusions

The last decade witnessed a growing interest in non-canonical DNA structures as well as their ligands as chemical biology tools operating at the genomic level. Unfortunately, in many cases, the conclusion about the mechanism of action of such ligands, namely via interaction with or stabilization of a given higher-order DNA structure, is based only on weak in vitro evidence of highly affine or selective binding. This is particularly important in the field of i-DNA, where reference ligands or standardized assays to access the affinity and selectivity of putative ligands are not yet available. In this regard, we re-examined the synthesis and i-DNA binding properties of **IMC-48**, one of the first compounds described as an i-DNA ligand almost a decade ago [20]. We delineated a straightforward and efficient synthesis of this compound from widely available cholesterol, with a global yield of 25% over four steps, and unambiguously confirmed its structure by X-ray crystallography. Remarkably, three biophysical technics, namely BLI, thermal, and chemical denaturation, point out to a very weak interaction of **IMC-48** with its cognate sequence *bcl-2* and other i-motifs (with *K*_D_ ≥ 50 µM), and the absence of a significant stabilization effect against thermal or chemical (i.e., increase of pH up to neutral) denaturation. Importantly, our results do not refute the ensemble of the data presented in the original report [20,21]; instead, they call for an alternative explanation for the biological activity of **IMC-48** (including downregulation of *bcl-2*), most likely unrelated to the stabilization of i-DNA in the *bcl-2* promoter. Transcriptional regulation is an incredibly complex process involving multiple protein and RNA partners [35], many of which may act as efficient druggable targets. Thus, careful in vitro characterization of interaction of a putative drug with its putative biological target(s) is a crucial and indispensable step to confirm (or disregard) the proposed mechanism of action.

## 4. Experimental Section

*Materials and Instruments*. Reactions were monitored by thin layer chromatography (TLC) using commercial alumina-backed silica gel plates. TLC spots were viewed by heating the plate after treatment with vanillin or potassium permanganate. Unless otherwise noted, all reagent-grade chemicals and solvents were obtained from commercial suppliers and were used as received. Chromatography purifications were performed by column chromatography using Silica Gel 60 (40–60 mesh). Melting points were obtained using a heating rate of 1 °C/min and are uncorrected. Infrared spectra (IR) were performed on a Fourier transform infrared spectrometer using ATR (Attenuated Total Reflexion) and the data are reported in reciprocal centimeters (cm^−1^). ^1^H-NMR spectra (400 MHz and 500 MHz) and ^13^C-NMR spectra (75 MHz) were recorded on Avance III 400 (Bruker) and Avance III 500 (Bruker) spectrometers. Low Resolution Mass Spectra (LRMS) were recorded on an ion-trap spectrometer (ESI). HRMS were recorded on a Waters XEVO^®^ G2-S QTof apparatus (ESI).

### 4.1. Crystal Structure Analysis

All crystals were selected, damped in a paraffin mixture, mounted on a nylon cryo-loop, then centered on a Bruker-AXS-enraf-nonius KappaAPEXII goniometer equipped with a high brilliance micro-source. The data was collected with φ and ω scans then integrated and corrected for Lorentz and polarization effects using the EVAL14 software. Final cell parameters were obtained post-refining the whole data. The data was then reintegrated and corrected for absorption using the SADABS program and finally merged with the software XPREP. The structure was solved by direct methods and refined by full-matrix least square methods with, respectively, the SHELXT-2016 and SHELXL-2013 programs implemented in Olex2 software. C, Cl, I, N, and O atoms were refined with anisotropic thermal parameters. H atoms were set geometrically, riding on the carrier atoms, with isotropic thermal parameters.

**5α-cholestan-3β-ol 1:** Crystal data for C_28_H_51_O_1.5_ triclinic, P1. a (Å) = 7.6990(15), b (Å) = 9.751(2), c (Å) = 34.975(7), α (°) = 86.37(3), β (°) = 86.94(3), γ (°) = 87.84(3), V (Å^3^) = 2615.1(9), Z = 4, D (g·cm^−3^) = 1.046. λ (Å) = 0.71073. F(000) = 924. μ (mm^−1^) = 0.062. T (K) = 200. Θ range (°) = 1.753–27.5. Measured, unique and used reflections: 70,144, 23,333 and 16,803. [R(int) = 0.0807]. 1295 parameters. R(1)[I > 2σ(I)] = 9.53%. WR(2) = 24.66%. G. O. F. = 1.047. N° CCDC 2218576.

**3α,5α-chloroacetylaminocholestane 5:** Crystal data for C_29_H_50_ClNO monoclinic, space group P2_1_. a (Å) = 9.2251(18), b (Å) = 18.147(4), c (Å) = 16.847(3), β (°) = 93.65(3), V (Å^3^) = 2814.6(10), Z = 4, D (g·cm^−3^) = 1.095. λ (Å) = 0.71073. F(000) = 1024. μ (mm^−1^) = 0.156. T (K) = 200. Θ range (°) = 2.423–25.0. Measured, unique and used reflections: 46,875, 9773, and 7178. [R(int) = 0.0777]; 636 parameters R(1)[I > 2σ(I)] = 5.42%. WR(2) = 9.78%. G. O. F. = 1.208. N° CCDC 2218577.

**IMC-48:** Crystal data for C_35_H_63_IN_2_O monoclinic, space group P2_1_. a (Å) = 13.198(3), b (Å) = 12.062(2), c (Å) = 22.656(5), β (°) = 95.19(3), V (Å^3^) = 3591.8(12), Z = 4, D (g·cm^−3^) = 1.211. λ (Å) = 0.71073. F(000) = 1392. μ (mm^−1^) = 0.918. T (K) = 200. Θ range (°) = 1.549–25.0. Measured, unique and used reflections: 67,163, 12,623, and 9091; [R(int) = 0.0851]; 847 parameters; R(1)[I > 2σ(I)] = 6.19%. WR(2) = 10.15%. G. O. F. = 1.193. N° CCDC 2218578.

### 4.2. Synthesis

**5α-cholestan-3β-ol 1:** PtO_2_ (199.9 mg, 6.8 mol%) and perchloric acid (200 µL, cat.) were added to a cholesterol (5 g, 12.95 mmol) solution in 100 mL of dry ethyl acetate. The flask was then flushed with hydrogen and kept under a hydrogen atmosphere for 2 h. The TLC (eluted with pentane/ethyl acetate: 7/3), revealed using vanillin (both starting material and final product have the same *r. f.*; however, the coloration is not the same after revelation), showed that the reaction was complete. The mixture was filtered over celite, washed with ethyl acetate, and the solvents evaporated under reduced pressure. A silica gel column chromatography was performed to isolate 0.68 g of acetylated 5α-cholestan-3β-ol (1.6 mmol, 12%) and 4.4 g of 5α-cholestan-3β-ol **1** (11.3 mmol, 87%) as white solids. 5α-cholestan-3β-ol **1** could be recrystallized by slow evaporation of AcOEt and gave X-ray suitable crystals. ^1^H-NMR (400 MHz, CDCl_3_): δ = 3.50–3.68 (m, 1H), 1.91–2.00 (m, 1H), 0.73–1.90 (m, 30H), 0.90 (d, 3H, *J* = 6.6 Hz), 0.86 (d, 6H, *J* = 6.1 Hz), 0.80 (s, 3H), 0.64 (s, 3H), 0.57–0.66 (m, 1H). ^13^C-NMR (75 MHz, CDCl_3_): δ = 71.4, 56.5, 56.3, 54.4, 44.9, 42.6, 40.1, 39.5, 38.2, 37.0, 36.2, 35.8, 35.5, 35.4, 32.1, 31.5, 28.7, 28.2, 28.0, 24.2, 23.8, 22.8, 22.5, 21.3, 18.7, 12.3, 12.1. IR: 3381, 2930, 2867, 2843, 1467, 1443, 1381, 1171, 1133, 1079, 1040, 959, 929, 735 cm^−1^. M.p.: 120–121 °C (lit.: 141–142 °C).

**3α,5α-5-chloroacetylaminocholestane 5:** Mg(HSO_4_)_2_ (48.7 mg, 1 eq.) [36] was added to a solution of 5α-cholestan-3β-ol **1** (86.5 mg, 0.223 mmol) in 0.9 mL of chloroacetonitrile and the reaction mixture was stirred at 90 °C for 6 h. The solvent was removed under reduced pressure and 5 mL of water were added to the residue. The organic phase was extracted with Et_2_O, the combined organic phases dried on magnesium sulfate, filtered, and evaporated. The residue was purified by flash chromatography on silica gel (pentane/AcOEt: 90/10 to 85/15), affording a first fraction of 25.0 mg of a mixture of diastereoisomers (**5a**/**5b**: 1/0.4), and a second fraction of 20.5 mg of pure **5a** (total 45.5 mg of both diastereoisomers, 44% global yield). Pure **5a** could be crystallized by slow evaporation of AcOEt and gave X-ray suitable crystals, confirming its 5α stereochemistry. ^1^H-NMR (400 MHz, CDCl_3_): δ = 6.88 (bs, 1H), 4.13 (bs, 1H), 4.04 (s, 2H), 1.93–2.01 (m, 1H), 0.76–1.87 (m, 29H), 0.89 (d, 3H, *J* = 6.6 Hz), 0.85 (dd, 6H, *J* = 6.5, 1.5 Hz), 0.81 (s, 3H), 0.66–0.74 (m, 1H), 0.64 (s, 3H). ^13^C-NMR (75 MHz, CDCl_3_): δ = 154.8, 56.5, 56.3, 54.5, 45.3, 43.0, 41.1, 39.9, 39.5, 37.3, 36.2, 36.1, 35.8, 35.4, 33.2, 32.6, 31.9, 28.4, 28.2, 28.0, 25.8, 24.1, 23.8, 22.8, 22.5, 20.8, 18.6, 12.1, 11.4. IR: 3259, 3076, 2924, 2864, 1686, 1647, 1554, 1464, 1455, 1444, 1381, 1363, 1231, 1168, 962, 923, 786, 693 cm^−1^. M.p.: 192–193 °C (lit.: 202 °C). HRMS (ESI) calcd. for C_29_H_51_ClNO^+^: 464.3654, found: 464.3633.

**3α,5α-5-piperidinoacetylaminocholestane 6:** 126 µL of piperidine (3 eq.) was added to 3α,5α-5-chloroacetylaminocholestane **5** (196.6 mg, 0.425 mmol) in 3.6 mL of dry EtOH at room temperature. The mixture was stirred at reflux for 4 h. The reaction mixture was then cooled down to room temperature, the solvent was evaporated, and the residue was treated with sat. aq. NaHCO_3_ solution and Et_2_O. The phases were separated, and the aqueous phase extracted twice with Et_2_O. The combined organic phases were dried on magnesium sulfate, filtered, and evaporated to provide a light-yellow solid (224.2 mg, quant. yield). ^1^H-NMR (400 MHz, CDCl_3_): δ = 7.74 (bd, 1H, *J* = 7.8 Hz), 4.08–4.15 (m, 1H), 2.92 (s, 2H), 2.38–2.53 (m, 4H), 1.94–2.02 (m, 1H), 0.77–1.87 (m, 36H), 0.90 (d, 3H, *J* = 6.6 Hz), 0.85 (dd, 6H, *J* = 6.6, 1.5 Hz), 0.81 (s, 3H), 0.63–0.67 (m, 1H), 0.65 (s, 3H). ^13^C-NMR (75 MHz, Acetone-d6): δ = 169.5, 62.4, 56.6, 56.3, 55.1, 54.9, 43.9, 42.6, 41.3, 40.0, 39.5, 36.2, 36.1, 35.7, 35.5, 33.6, 33.1, 32.2, 28.5, 28.2, 28.0, 26.5, 26.2, 24.1, 23.8, 23.7, 22.8, 22.5, 20.8, 18.7, 12.2, 11.5. IR: 3253, 2926, 2849, 1683, 1656, 1560, 1509, 1440, 1378 cm^−1^. M.p.: 124–125 °C (lit.: 134–135 °C). HRMS (ESI) calcd. for C_34_H_61_N_2_O^+^: 513.4778, found: 513.4767.

**IMC-48:** Iodomethane (7 µL, 1.1 eq.) was added to 3α,5α-5-piperidinoacetylaminocholestane **6** (55.2 mg, 0.108 mmol) in 1 mL of dry MeOH in an open sealed tube at room temperature. The tube was closed and heated at 70 °C for 48 h. The reaction mixture was then cooled down to room temperature, and cold Et_2_O was added. The solid was filtered and washed with cold Et_2_O, then dried under vacuo to provide 47.0 mg of **IMC-48** as a white solid (0.072 mmol, 67%). **IMC-48** could be recrystallized by slow evaporation of ethanol affording X-ray suitable crystals which confirmed the expected structure. ^1^H-NMR (400 MHz, DMSO-d6): δ = 8.40 (bd, 1H, *J* = 7.6 Hz), 4.11 (s, 2H), 3.97–4.03 (m, 1H), 3.47–3.55 (m, 2H), 3.37–3.47 (m, 2H), 3.22 (s, 3H), 1.92–2.05 (m, 2H), 0.77–1.87 (m, 34H), 0.90 (d, 3H, *J* = 6.6 Hz), 0.85 (dd, 6H, *J* = 6.6, 2.5 Hz), 0.78 (s, 3H), 0.68–0.75 (m, 1H), 0.64 (s, 3H). ^13^C-NMR (75 MHz, DMSO-d6): δ = 162.2, 61.3, 60.5, 56.3, 55.7, 53.8, 44.5, 42.2, 39.8, 38.9, 35.6, 35.5, 35.2, 34.9, 32.3, 32.2, 31.7, 28.1, 27.8, 27.4, 25.5, 25.4, 23.8, 23.2, 22.7, 22.4, 20.6, 20.3, 19.3, 18.5, 11.9, 11.2. IR: 3232, 3040, 2930, 2864, 2846, 1665, 1536, 1464, 1440, 1363, 1252, 1171, 941, 905 cm^−1^. M.p.: 235–236 °C (lit.: 267 °C). HRMS (ESI) calcd. for C_35_H_63_N_2_O^+^: 527.4935, found: 527.4921.

*Oligonucleotide sequences.* The oligonucleotides used in this study were purchased from IDT and a PEG-biotin moiety was attached at the 3’ extremity for subsequent surface anchoring for BLI analysis. The corresponding non-biotinylated sequences were used for CD analysis. Before each experiment, the oligonucleotides were annealed by heating at 90 °C for 5 min prior to a slow cooling to room temperature over one hour to ensure their folding into i-motif or hairpin structures.

*Bio-layer interferometry (BLI) measurements.* BLI sensors coated with streptavidin (SA sensors) were purchased from Forte Bio (Sartorius). Prior to use, they were immerged 10 min in buffer before functionalization to dissolve the sucrose layer. Then the sensors were dipped for 15 min in 100 nM biotinylated DNA sequences containing solutions and rinsed in buffer solution (50 mM Tris-HCl, 50 mM KCl, 2% DMSO, and 0.05% *v/v* surfactant P20 at pH 5.5 and 7.5) for 10 min. The functionalized sensors were next dipped in solutions containing **IMC-48** at different concentrations for 500 s interspersed by a rinsing step in the buffer solution during 500 s. Reference sensors without DNA immobilization were used to subtract the non-specific adsorption on the SA layer. No regeneration was performed after each recognition and the same sensor was dipped in all the solutions containing the various concentrations of **IMC-48**. The dissociation equilibrium constant, *K_D_*, was determined by the fitting of the Langmuir isotherm (equilibrium response versus **IMC-48** concentrations). The reported values are the means of representative independent experiments, and the errors provided are standard deviations from the mean. Each experiment was repeated at least three times.

*CD melting experiments.* The thermal denaturation profiles were recorded on a Jasco J-1500 CD spectrophotometer equipped with a Peltier-controlled multicell holder. Prior to measurements, samples containing 2.5 µM oligonucleotides in aqueous buffer (10 mM LiAsMe_2_O_2_, 100 mM KCl, pH 5.5; total volume 1 mL) were annealed at 90 °C for 5 min, cooled to room temperature, and supplemented with **IMC-48** (final concentration: 20 µM) or DMSO control (20 µL) and transferred into semi-micro quartz cuvettes (Hellma 114B-QS, 10 mm light path). The measurement cycle involved heating the samples from 20 to 90 °C at a rate of 0.2 °C min^−1^, followed by cooling to 20 °C at the same rate. The ellipticity was monitored at λ = 285 nm (bandwidth: 2 nm, integration time: 2 s). The temperatures of melting transitions (*T*_m_) were determined from the Boltzmann fits of plots of the ellipticity versus temperature. The experiments were performed in triplicate.

*CD-potentiometric titrations.* Chemical denaturation profiles were recorded on a Jasco J-1500 CD spectrophotometer equipped with a Peltier-controlled multicell holder, magnetic stirrer, automatic titration unit (Jasco ATS-530), and InLab micro pH electrode (Mettler Toledo). Prior to measurements, samples containing 2.5 µM oligonucleotides in aqueous buffer (10 mM LiAsMe_2_O_2_, 100 mM KCl, pH adjusted to 4; total volume: 2 mL) were annealed at 90 °C for 5 min, cooled to room temperature, supplemented with **IMC-48** (final concentration: 20 µM) or DMSO control (20 µL), and transferred into quartz cuvettes (1 cm × 1 cm). The measurement cycle consisted of 25 additions of NaOH (1 M, 1 µL per aliquot) followed by 25 additions of HCl (1 M, 1 µL per aliquot) to perform the whole titration. The temperature was maintained at 20 °C, the cuvette content was stirred, and pH was recorded during the whole experiment. The ellipticity was monitored at λ = 285 nm (bandwidth: 2 nm, integration time: 2 s). The transition pH values were determined from the Boltzmann fits of plots of the ellipticity versus pH. The titrations were performed in duplicate.

## Figures and Tables

**Table 1:** Oligonucleotide sequences used in the present study.

Acronym	Sequence (5′ → 3′)
*c-myc*	TCCCCACCTTCCCCACCCTCCCCACCCTCCCCA
*HRAS*	CGCCCGTGCCCTGCGCCCGCAACCCGA
*Bcl-2*	CAGCCCCGCTCCCGCCCCCTTCCTCCCGCGCCCGCCCCT
*Htelo-C*	TAACCCTAACCCTAACCCTAACCCTAA
*ssDNA*	GGCATAGTGCGTGGGCG
*HP-GC*	CGCGCGCGTTTTCGCGCGCG
*DAP-Mut*	CTACCGCTAACGCCTTCGCTCTCGCTTTC

**Table 2:** Effect of **IMC-48** on thermal (∆*T*_m_) and chemical (∆pH_T_) stability of i-DNA from CD-monitored denaturation experiments.

Sequence	∆*T*_m_/°C *^a^*		∆pH_T_ *^b^*	
	Denaturation	Renaturation	Denaturation	Renaturation
*HRAS*	−1.2 ± 1.1	−0.5 ± 1.1	−0.02 ± 0.15	−0.04 ± 0.06
*bcl-2*	0.2 ± 0.4	0.2 ± 0.9	0.01 ± 0.08	−0.02 ± 0.03
*HTelo-C*	−1.2 ± 0.5	−0.7 ± 0.9	0.10 ± 0.04	−0.06 ± 0.08
*c-myc*	−0.5 ± 0.1	−1.1 ± 1.2	0.03 ± 0.06	−0.11 ± 0.06

*^a^* Ligand-induced shifts of melting temperature in thermal denaturation experiments; conditions: *c*(oligo) = 2.5 µM, *c*(**IMC-48**) = 20 µM in 10 mM LiAsO_2_Me_2_, 100 mM KCl buffer, and pH 5.5. ∆*T*_m_ values were determined from both heating (denaturing) and cooling (renaturing) ramps. Data are means ± SD from three independent experiments. *^b^* Ligand-induced shifts of transition pH determined from acid-base titrations performed in the absence and in the presence of the ligand; conditions: *c*(oligo) = 5 µM, *c*(**IMC-48**) = 20 µM in 10 mM LiAsO_2_Me_2_, 100 mM KCl buffer, *T* = 20 °C, and pH range: 5.0 to ~8. ∆pH_T_ values were determined both from alkali (denaturing) and acid (renaturing) titrations. Data are means ± SD from two independent titration experiments.

## Data Availability

Data could be sent upon demand to the corresponding authors.

## Supplementary Materials

The following supporting information can be downloaded at: https://www.mdpi.com/article/10.3390/molecules28020682/s1: Characterization of the different compounds: Figures S1–S13; CD spectra of the different studied sequences: Figure S14; BLI analysis: Figures S15 and S16; CD melting experiments: Figures S17 and S18.

## Sample Availability

Samples of the **IMC-48** are available from the authors upon request.
