# Supplementary material for: Novel Synthesis of IMC-48 and Affinity Evaluation with Different i-Motif DNA Sequences
Source: Molecules. 2023 Jan 10;28(2):682. doi: 10.3390/molecules28020682 (PMC9865601; doi:10.3390/molecules28020682)

Characterization of the different compounds

Figure S1. ^{1}H-NMR spectrum of 5α-cholestan-3β-ol **1.**

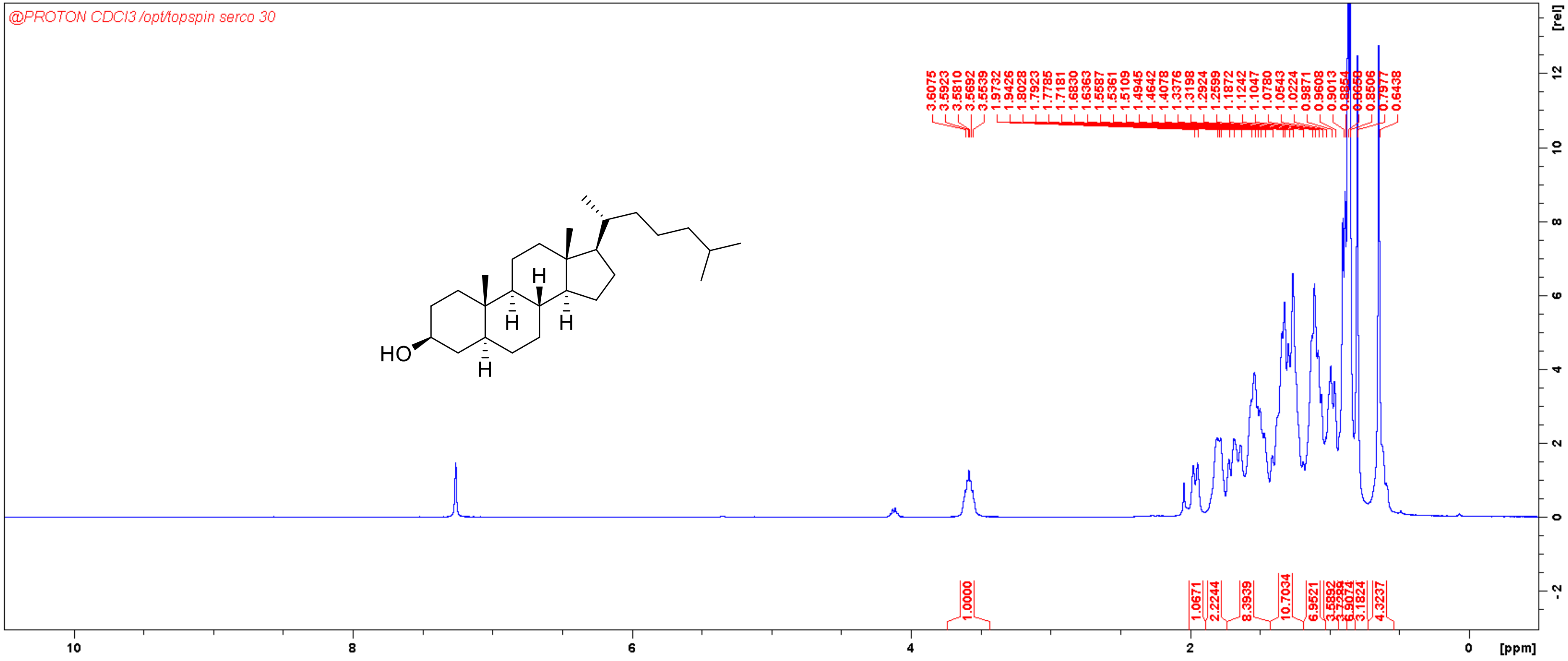

Figure S2. ^{13}C-NMR spectrum of 5α-cholestan-3β-ol **1.**

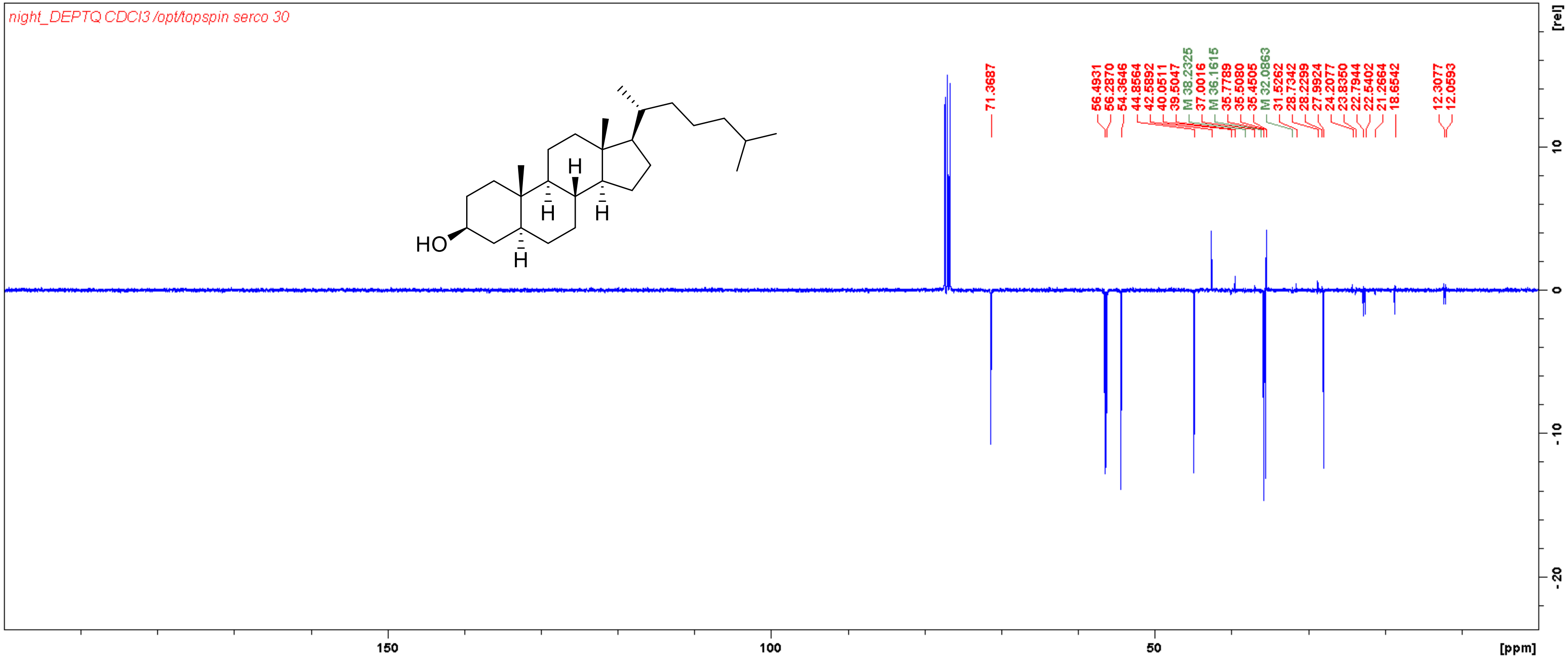

Figure S3. GC-MS analysis of 5α-cholestan-3β-ol **1.**

Qualitative Analysis Report

**Data Filename**	A_21245_02.D	**Sample Name**	befl002HF4
**Sample Type**		**Position**	3
**Instrument Name**		**User Name**	
**Acq Method**	AD_EI_CH2Cl2_300C.M	**Acquired Time**	9/2/2021 11:40:09 AM
**IRM Calibration Status**	Not Applicable	**DA Method**	AD_Method.m
**Comment**			

**Expected Barcode**		**Sample Amount**	
**Dual Inj Vol**	2	**TuneName**	etune20210902.u
**TunePath**	D:\MassHunter\GCMS\1\5977 \	**MSFirmwareVersion**	6.00.21
**Acquisition Time #2**	2021-09-02 09:40:09Z	**OperatorName**	
**RunCompletedFlag**	True	**Acquisition SW Version**	MassHunter GC/MS Acquisition B.07.01.1805 12-Mar-2014 Copyright © 1989-2014 Agilent Technologies, Inc.

User Chromatograms

Fragmentor Voltage **Collision Energy** 0 **Ionization Mode** Unspecified

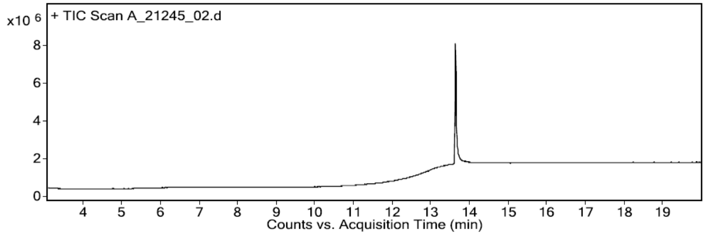

User Spectra

Collision Energy	Ionization Mode
0	Unspecified

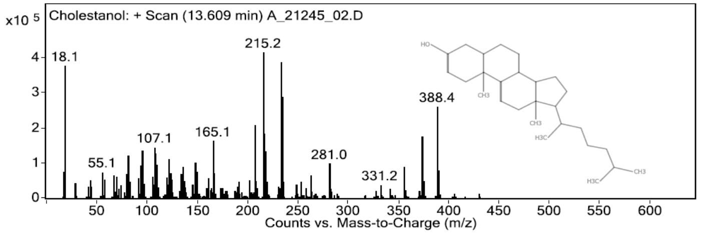

Spectrum Structure

Cholestanol

Qualitative Analysis Report

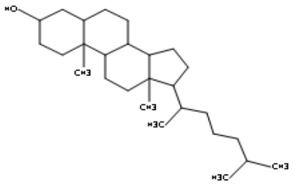

NIST Score : 85.2%

--- End Of Report ---

Figure S4. ^{1}H-NMR spectrum of 3α,5α-chloroacetylaminocholestane **5.**

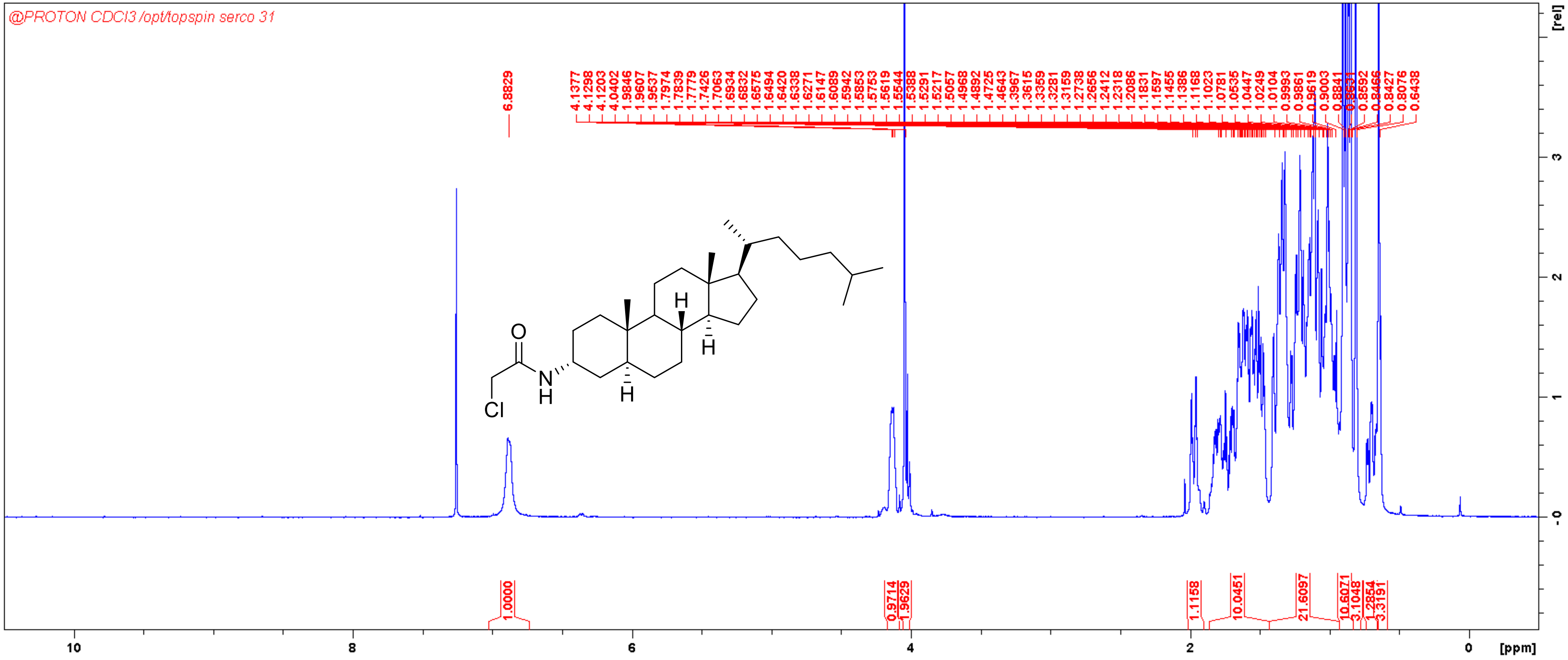

Figure S5. ^{13}C-NMR spectrum of 3α,5α-chloroacetylaminocholestane **5.**

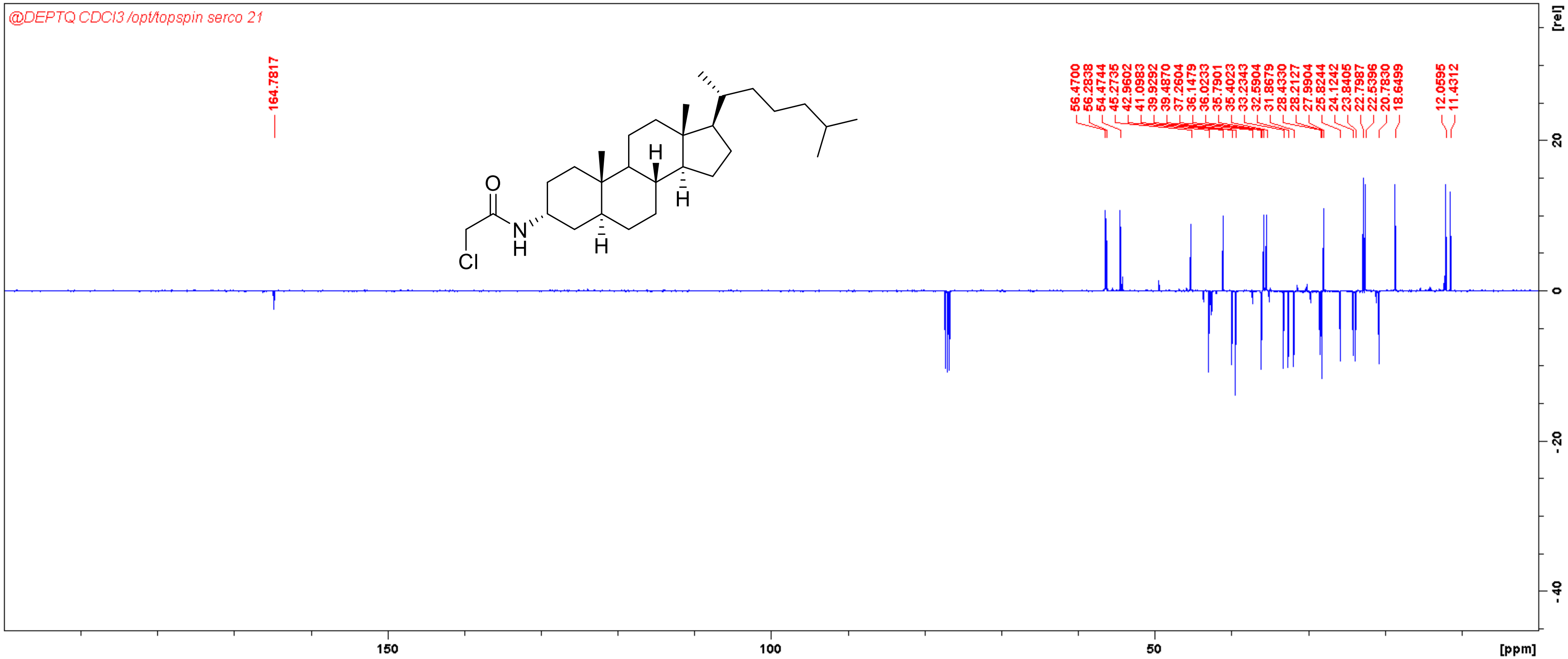

Figure S6. X-ray analysis of 3α,5α-chloroacetylaminocholestane **5**

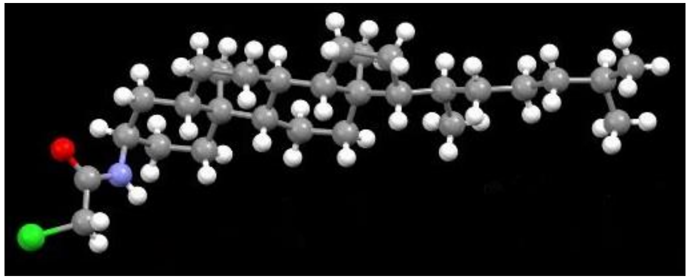

Figure S7. ^{1}H-NMR spectrum of 3α,5α-piperidinoacetylaminocholestane **6.**

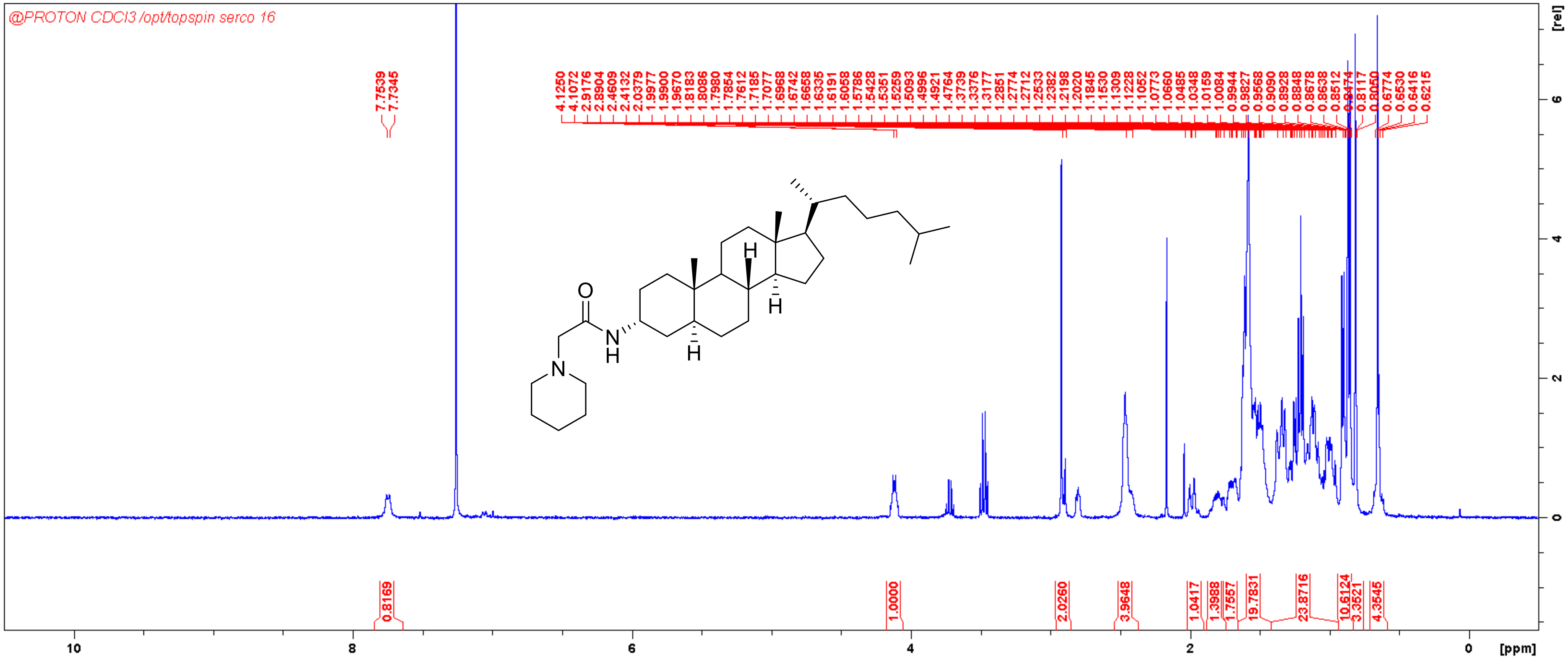

Figure S8. ^{13}C-NMR spectrum of 3α,5α-piperidinoacetylaminocholestane **6.**

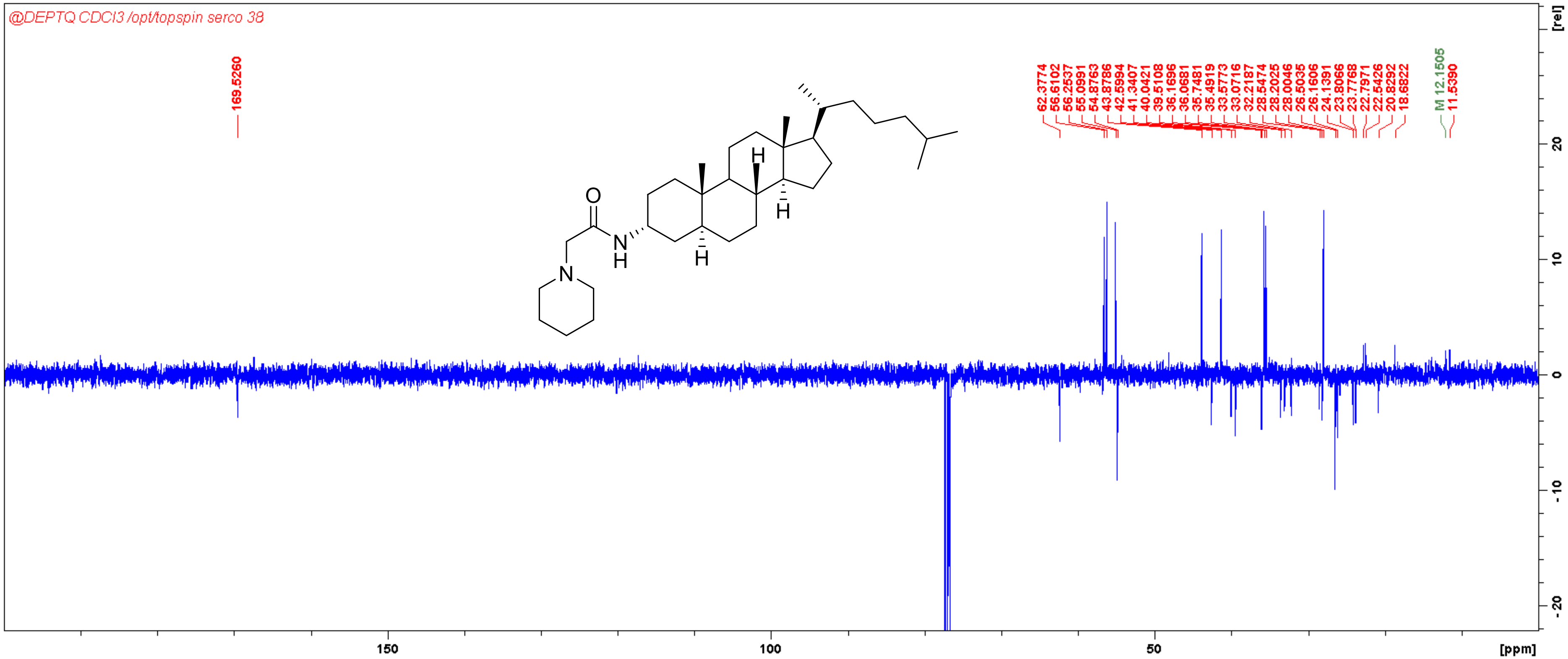

Figure S9. ^{1}H-NMR spectrum of **IMC-48.**

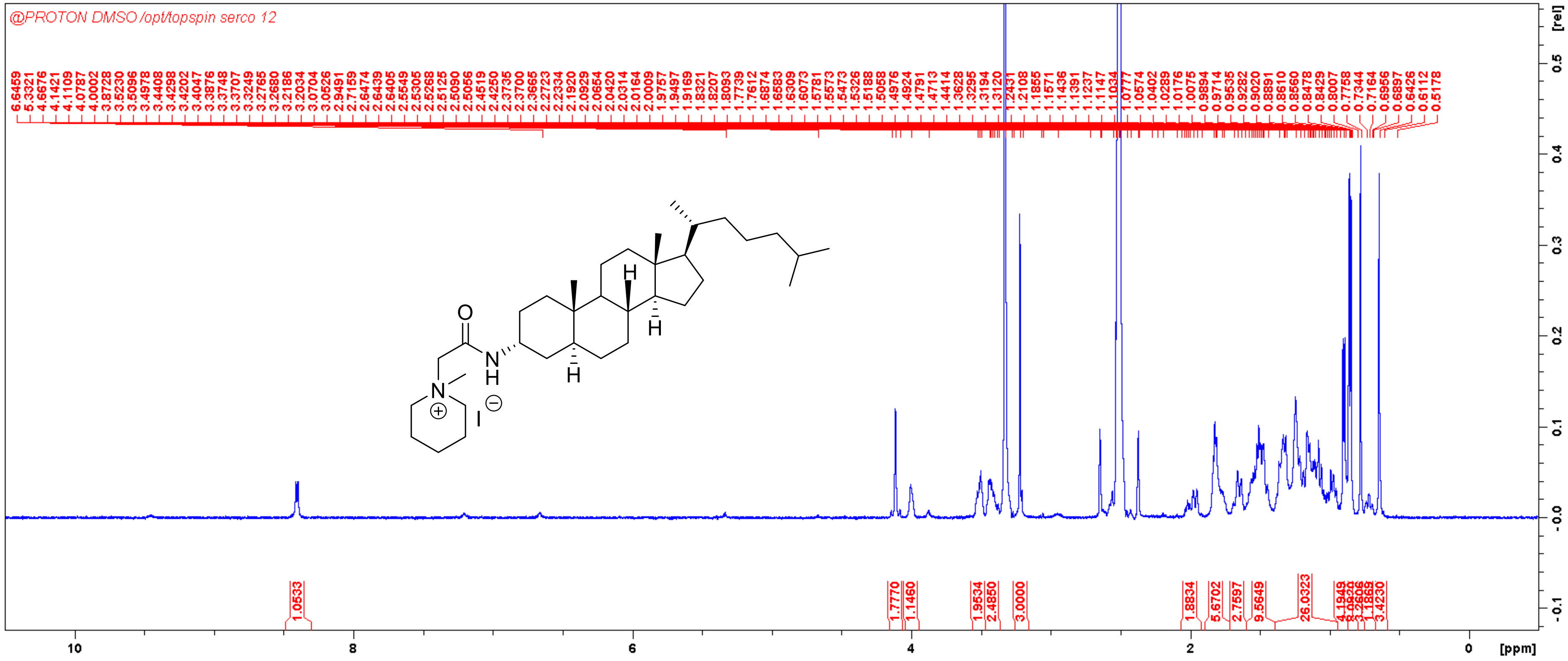

Figure S10. ^{13}C-NMR spectrum of **IMC-48**

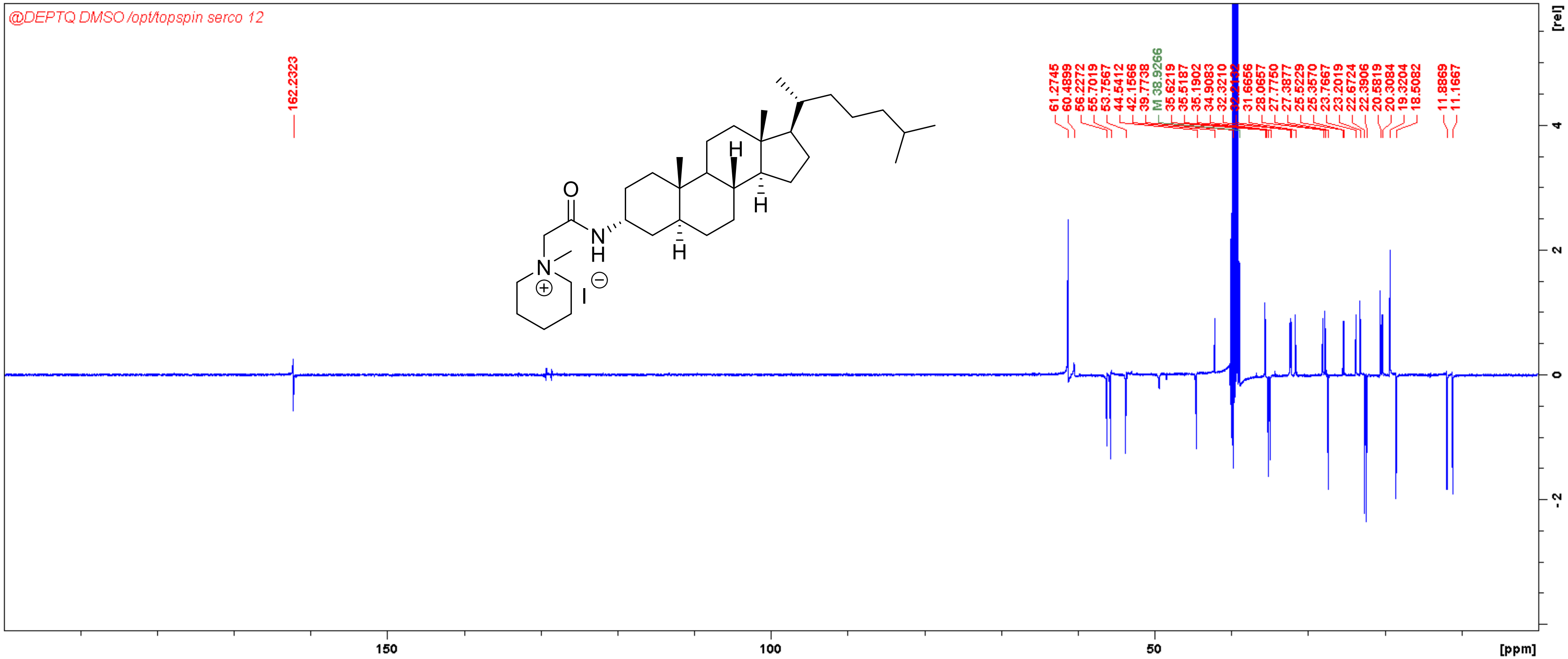

Figure S11. HPLC-MS analysis of **IMC-48**

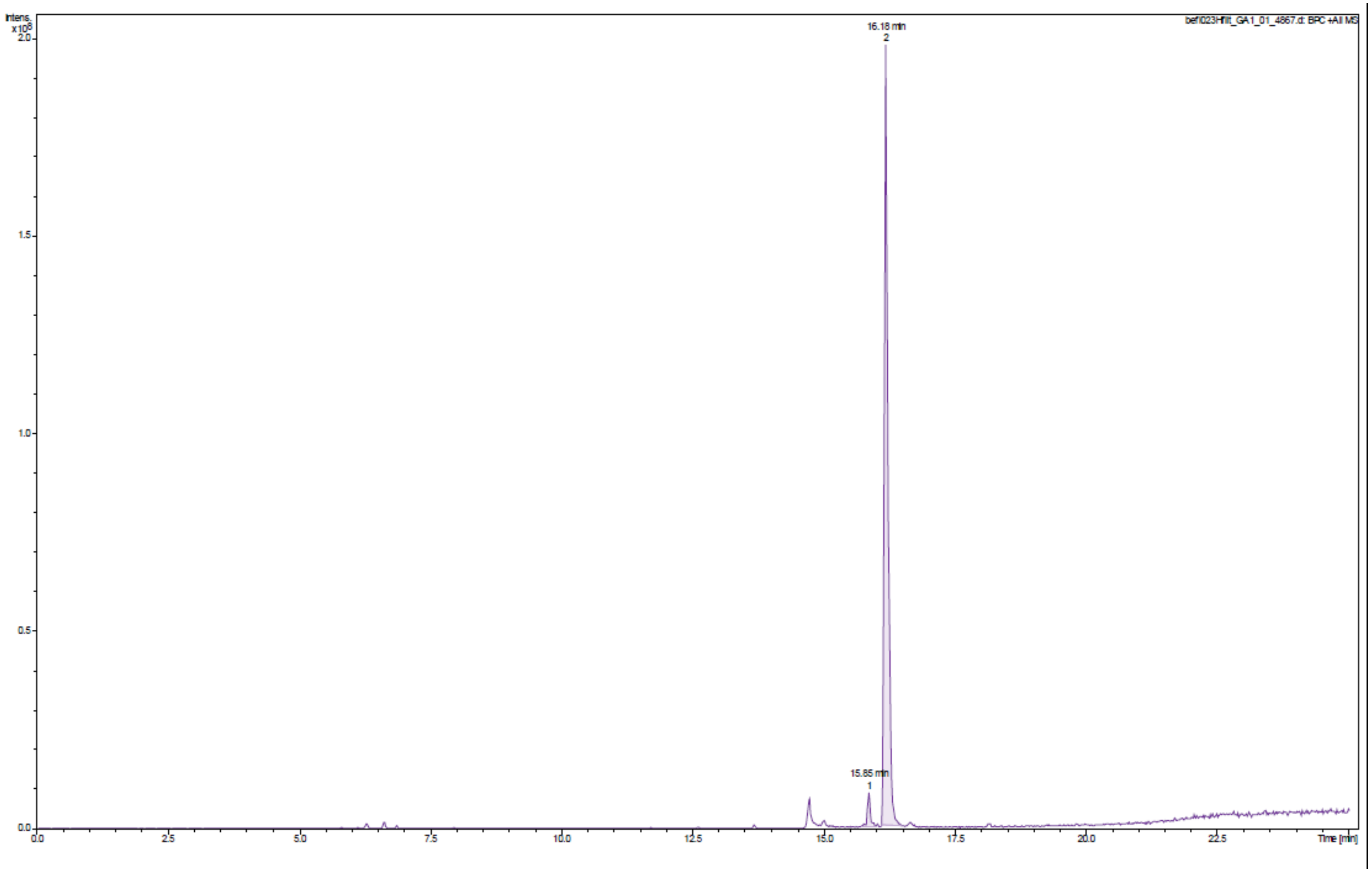

Figure S12. HPLC-MS analysis of **IMC-48**

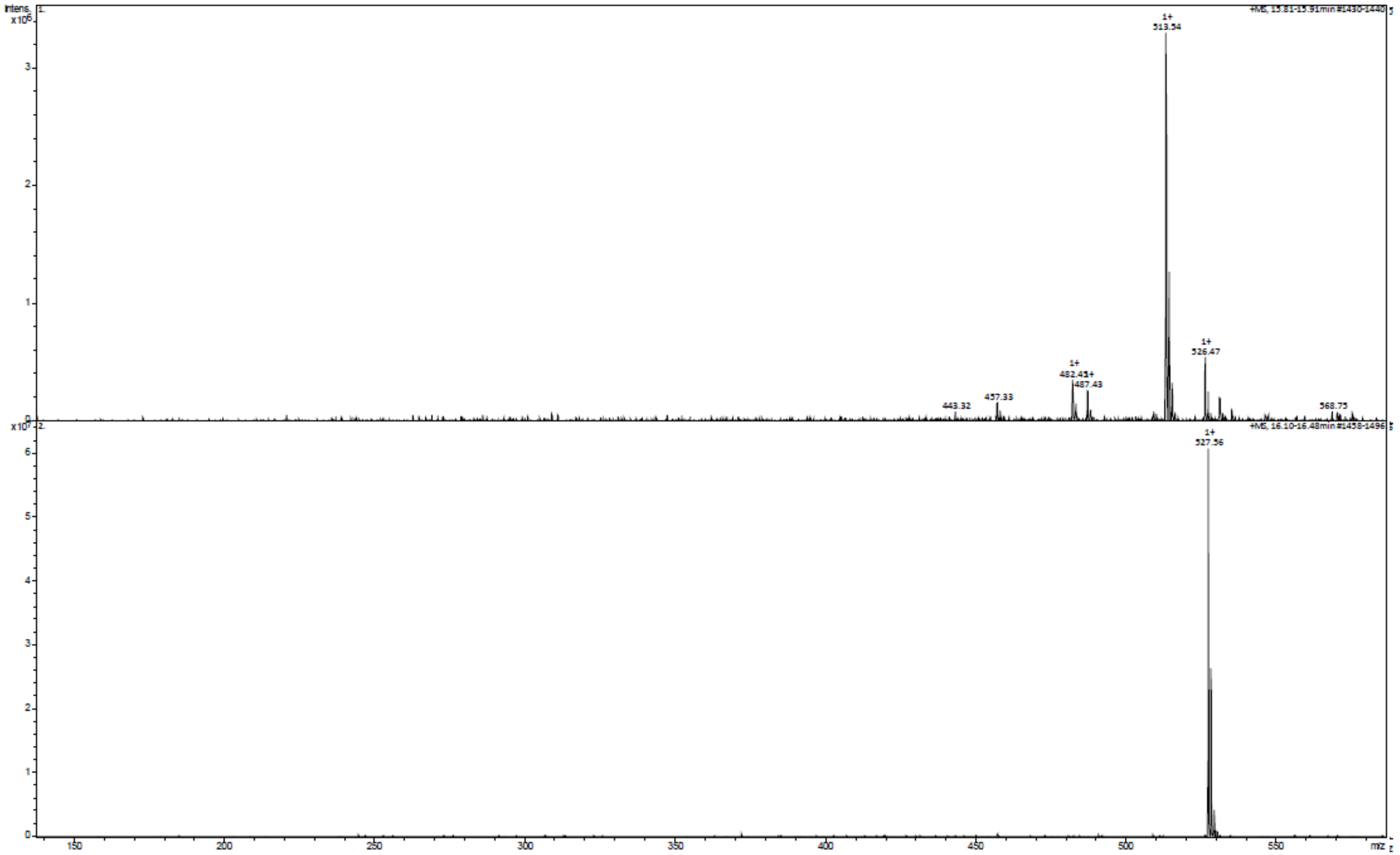

Figure S13. HPLC-MS of **IMC-48** (background down).

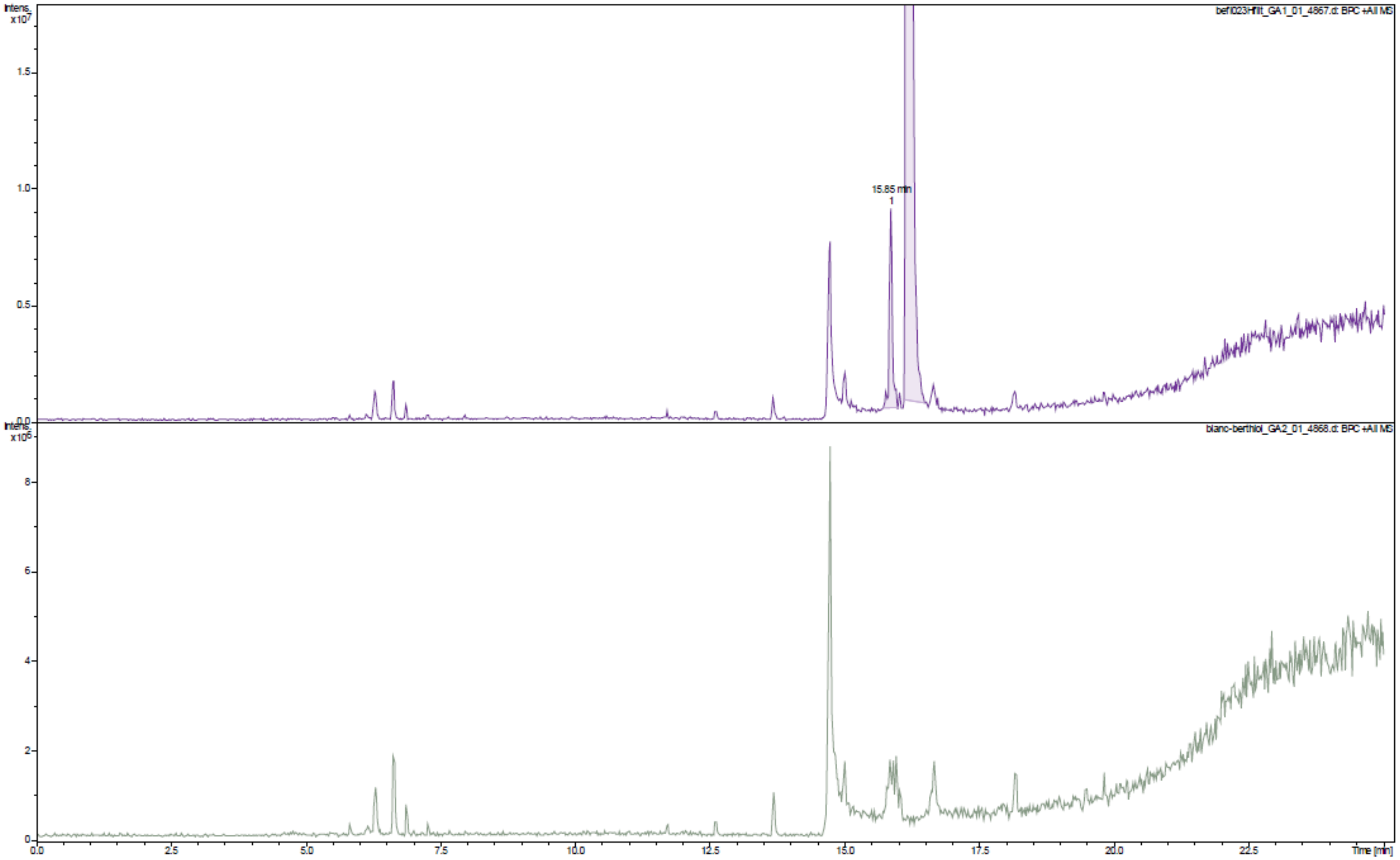

CD spectra.

Figure S14. CD analyses of the i-motif sequences at pH 5.5 (A) and 7.5 (B). *HRAS (*black*)*, *bcl-2 (*red*)*, *HTelo-C (*blue*)*, *C-myc (*green*)* and *DAP-Mut* (orange). Buffer: 50 mM TrisHCl, 50 mM KCl, 2 % DMSO at pH = 5.5 and 7.5

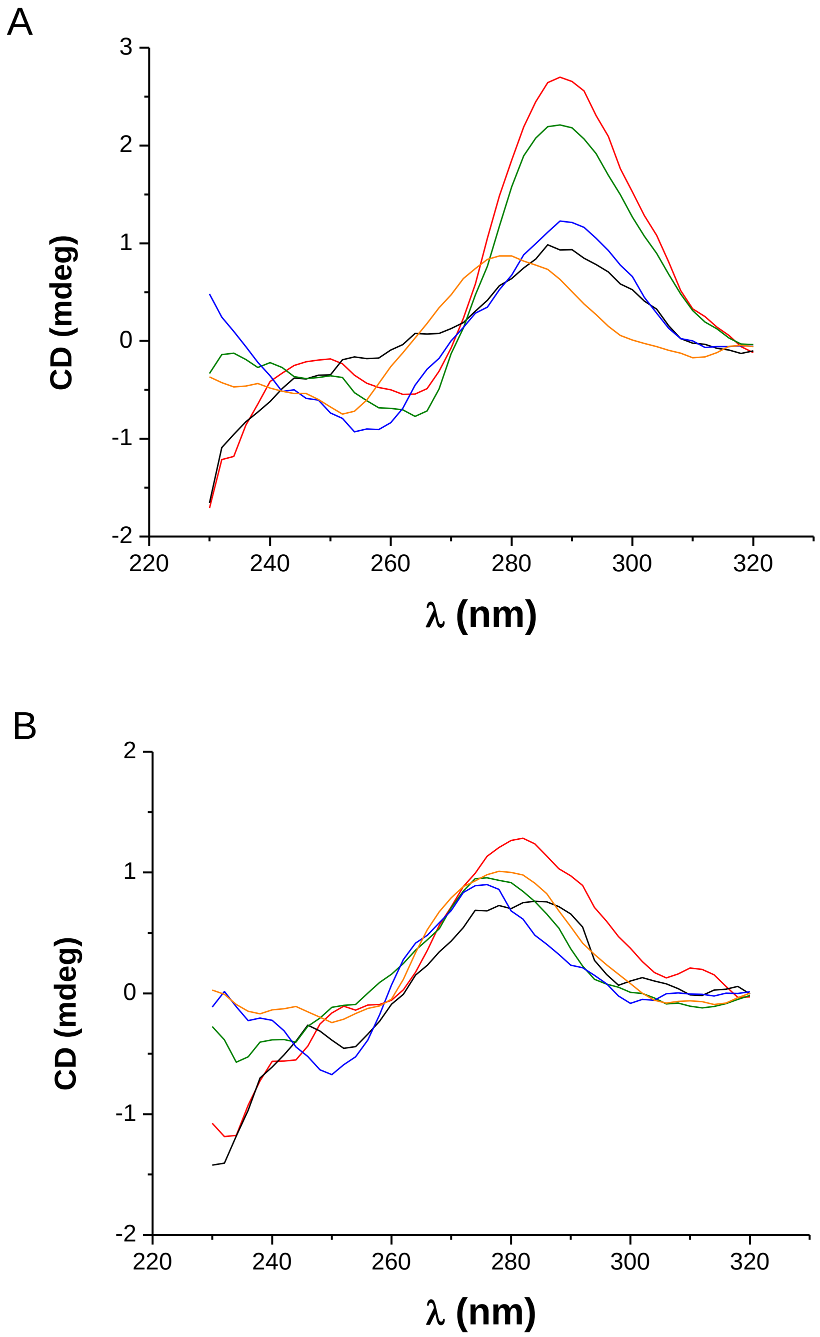

BLI analysis

Figure S15. Langmuir isotherm with the plateau value at the equilibrium at pH 5.5 (A) or pH 7.5 (B) for *HRAS (black)*, *bcl-2 (red)*, *HTelo-C* (blue), *c-myc (violet)*, HP GC (green), DAP-Mut (orange) and ssDNA (cyan). Concentration range: 0.1, 0.5, 1, 5, 10, 25, 50 and 100 µM.

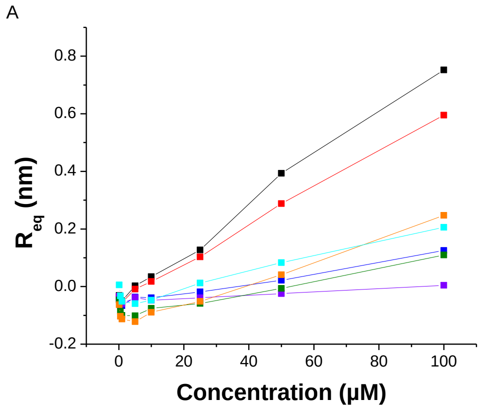

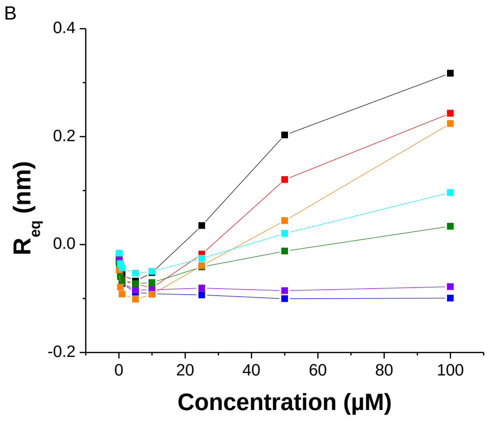

Figure S16. Sensorgrams recorded for the recognition of **IMC-48** with the control sequences: A) *HP GC* at pH 5.5, B) *DAP-Mut* at pH 5.5, C) *ssDNA* at pH 5.5, D) *HP GC* at pH 7.5, E) *DAP-Mut* at pH 7.5 and F) *ssDNA* at pH 7.5. Concentration range: 0.1 (blue), 0.5 (brown), 1 (green), 5 (dark cyan), 10 (black), 25 (orange), 50 (violet) and 100 µM (pink).

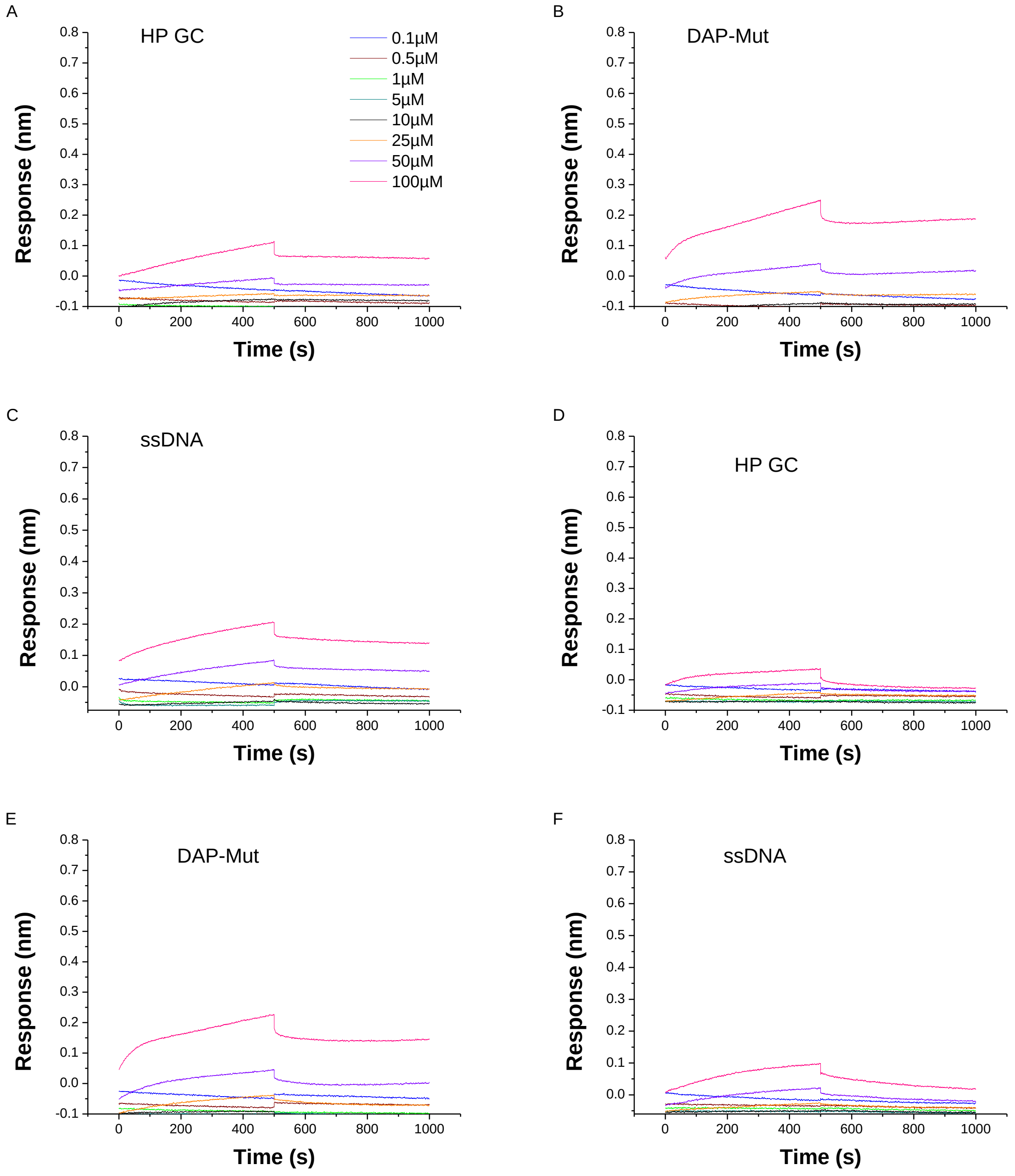

CD study

Figure S17. Circular dichroism spectra of A) *HRAS*, B) *bcl-2*, C) *HTelo-C* and D) *c-myc* at pH=7.2 in the absence (blue points) and in the presence of 20 µM of **IMC-48** (green points) and at pH=5.5 in the absence (red points) and in the presence of 20 µM of **IMC-48** (black points).

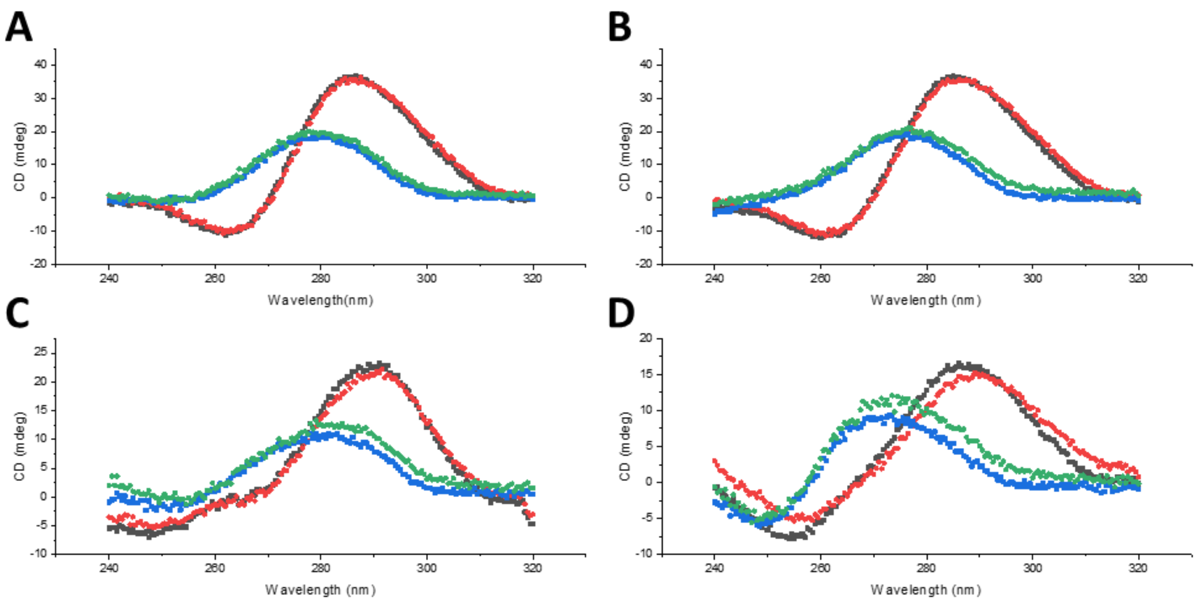

Figure S18. Representative thermal denaturation profiles of A) *HRAS*, B) *bcl-2*, C) *HTelo-C* and D) *c-myc* in the absence (black points) and in the presence of 20 µM of **IMC-48** (red points). Left panels: heating (denaturing) ramps, right panels: cooling (renaturing) ramps. The arrows indicate the denaturing/renaturing processes.

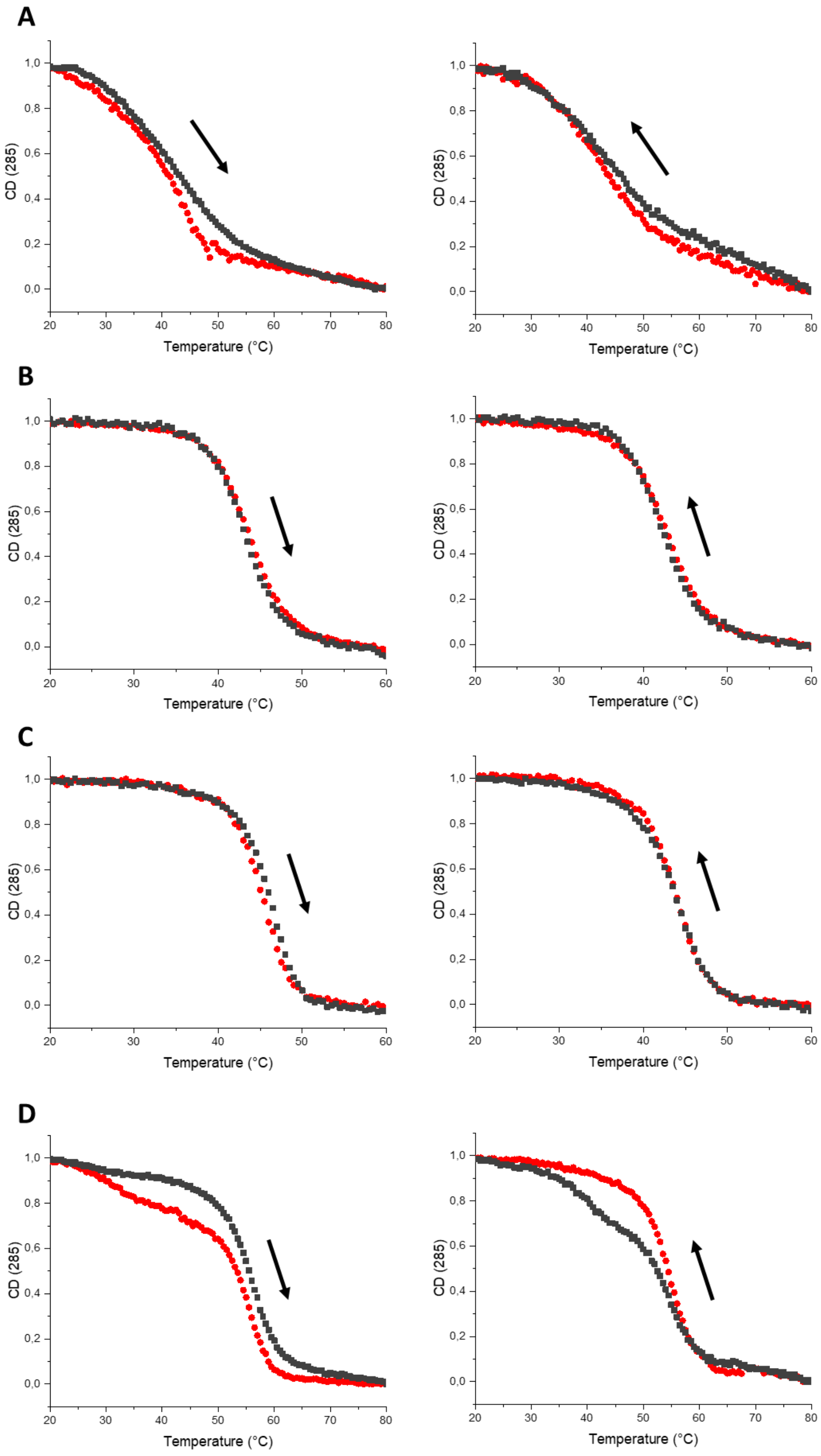

Supplement: Supplementary file 1 [file molecules-28-00682-s001.zip › molecules-2111740-supplementary.pdf]
